# Supplementary material for: Controlling light in complex media beyond the acoustic diffraction-limit using the acousto-optic transmission matrix
Source: Nat Commun. 2019 Feb 12;10:717. doi: 10.1038/s41467-019-08583-6 (PMC6372584; doi:10.1038/s41467-019-08583-6)
Supplement: Supplementary file 1 — Supplementary Information [file 41467_2019_8583_MOESM1_ESM.pdf]

Controlling light in complex media beyond the acoustic diffraction-limit using the acousto-optic transmission matrix

Katz et al.

Supplementary Information

Supplementary note 1: Experimental setup

A sketch of the optical experimental setup is given in Supplementary Figure 1. The scheme for the triggering and electronic connections is given in Supplementary Figure 2.

The optical setup is based on double-heterodyne detection scheme for acousto-optic tomography[1]. This detection scheme combines off-axis holography with phase shifting holography to provide sensitive detection of the weak ultrasonically modulated signal over the strong unmodulated background[2].

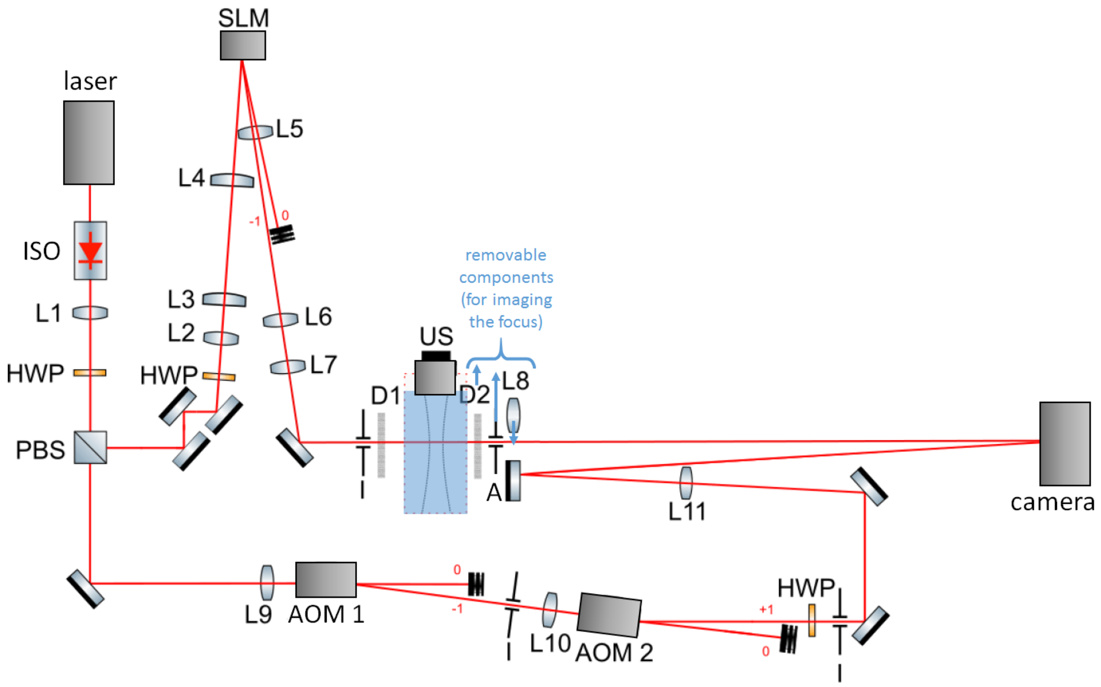

Supplementary Figure 1: Experimental set-up (ISO – Isolator, L# - lens, HWP – half wave plate, PBS – polarization beam splitter, AOM – acousto-optic modulator, I – iris, D# - diffuser, A – rectangular aperture, CCD – camera, US – ultrasound transducer, SLM – spatial light modulator)

The light source is a long-coherence continuous-wave infrared semiconductor laser. It is a compact (8×4×6cm) tunable extended cavity single longitudinal mode laser, centered at 810nm. The laser was provided by DTU Fotonik, Denmark (see acknowledgements). The compact laser can provide up to 1.5 Watt, without the need of an optical amplifier. 300mW average power was used in our experiments. The laser beam passes an isolator, is collimated by L1 and split to two arms of an interferometer by a polarization beam splitter. The relative powers in the two arms are controlled by a half wave plate. The beam paths in the signal and reference arms are as follows:

The beam at the signal arm is magnified using a telescope made up of lens L2 and two cylindrical lenses L3 and L4 (at different axes) to match the spatial light modulator (SLM) dimensions. The zero diffraction order of the light shaped by the SLM is cut, and a telescope made out of lenses L6 and L7 images the SLM on the first diffuser, D1, using the light from the first diffraction order. An iris, I, placed before the first diffuser controls the speckle size at the target plane. The light passes a water-filled cuvette, where the ultrasound beam is focused at. A second diffuser (D2) is placed at the output facet of the cuvette. The scattered light passes an aperture (A) that matches the speckle size on the camera to the camera resolution.

The reference arm passes two acousto-optic modulators, and is polarization rotated by a HWP to match the scattered light polarization at the signal arm. The reference arm is focused by L11 at the plane of the aperture placed after the exit of the second diffuser D2, and is reflected by a mirror to be combined at a small angle on the camera. To directly image the focal plane, the second diffuser D2 and the aperture, A, are removed and an imaging lens L8 is used to image the focal plane on the camera.

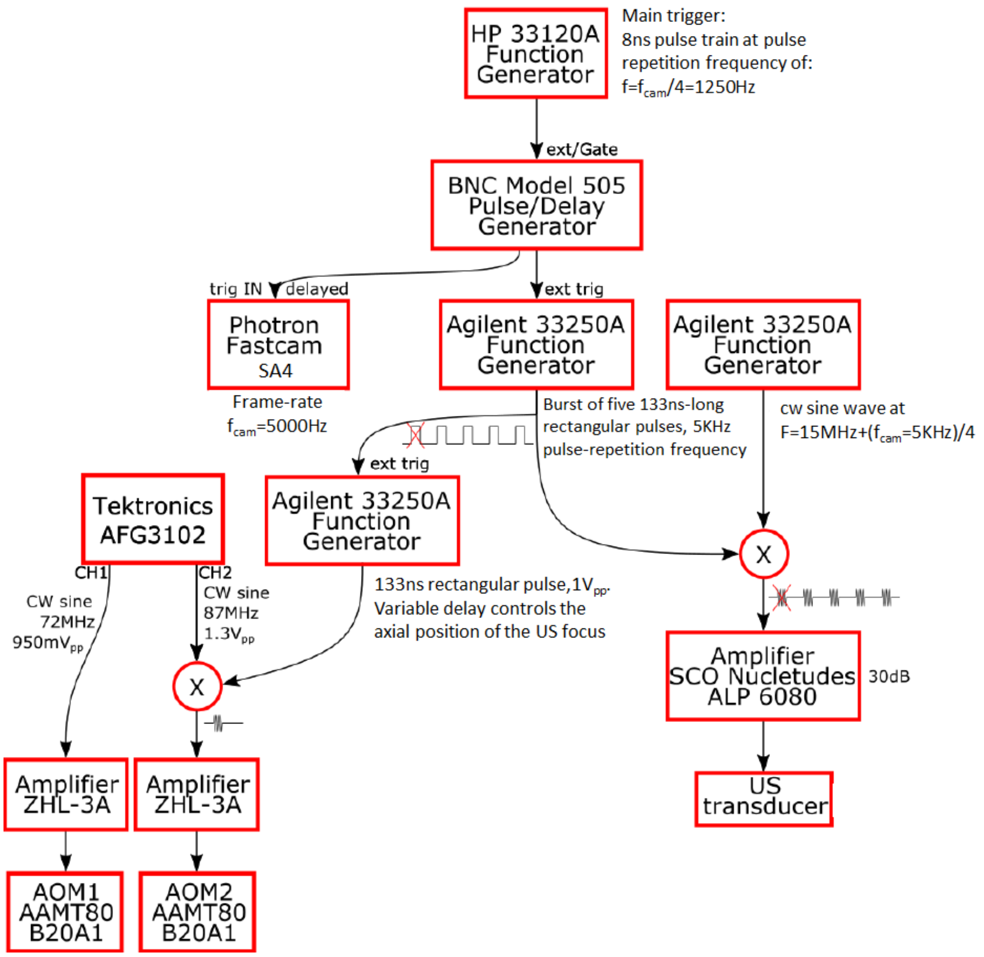

Supplementary Figure 2: Electronic connections and triggering scheme

Supplementary note 2: TRUE focusing as phase conjugation of an acousto-optic transmission matrix row

The acousto-optic transmission matrix (AOTM), T, describes the propagation of light from the SLM plane, through the ultrasound focus, and to the camera plane. As result of optical reciprocity, the matrix describing the propagation of light from the m-th camera pixel through the ultrasound focus and to the SLM plane is T^{T}. Thus, if light is injected into the medium from the mth pixel on the camera side, it will result in an output field at the SLM plane given by: $T^T E_{\mathrm{in,cam}}$, i.e. by the mth column of T^{T}. If this field is used for TRUE focusing, TRUE focusing will be obtained by displaying on the SLM the phase conjugate of this field, i.e. the phase conjugate of the mth column of T^{T}, which is the mth row of T, given by $(t_{m,*})^*$. Thus, displaying the mth row of the AOTM, T, is equivalent to performing TRUE-focusing for the light input from the mth camera pixel.

Supplementary note 3: Equivalence between the first singular vector of the AOTM and infinite iterations of iterative time-reversed ultrasound encoded (iTRUE) focusing

Here we analyze iterative TRUE (iTRUE)[3, 4] iterations under the AOTM framework. Following the definition of the AOTM as the relation between the input field to the medium and measured ultrasonically tagged field (Eq.1), the measured field on the camera in the first iTRUE iteration is given by:

$$E^{\text{out}}(f_{\text{o}} + f_{\text{US}}) = TE^{\text{in}}(f_{\text{o}}) \qquad (1)$$

In the first iterations this field is phase-conjugated and injected back into the medium at the original laser frequency. Due to optical reciprocity, the propagation of this phase-conjugated optical field back through the medium and through the ultrasonic focus is given by T^{T}. Thus, the optical field measured at the output in the first iTRUE iteration, which is the input field to the second iTRUE iteration is given by:

$$E^{\text{out,1}}(f_{\text{o}} + f_{\text{US}}) = T^{\text{T}}\left(TE^{\text{in}}(f_{\text{o}})\right)^{*} = T^{\text{T}}T^{*}\left(E^{\text{in}}(f_{\text{o}})\right)^{*} \qquad (2)$$

At the second iTRUE iteration, $E^{\text{out,1}}$ is phase-conjugated to provide:

$$E^{\text{in,2}} = \left(E^{\text{out,1}}\right)^{*} = \left(T^{\text{T}}T^{*}\left(E^{\text{in}}(f_{\text{o}})\right)^{*}\right)^{*} = (T^{\text{H}}T)E^{\text{in}}(f_{\text{o}}) \qquad (3)$$

Where T^{H} is the Hermitian conjugate of the matrix *T*.

Thus, the field conjugated at the $(2k)^{\text{th}}$ iTRUE iteration is given by:

$$E^{\text{in},2k} = (T^{\text{H}}T)^{k}E^{\text{in}} \qquad (4)$$

In ultrasound $T^{\text{H}}T$ was termed the 'time reversal operator' (TRO) [5, 6]. Theoretically, the tightest optical focus will be obtained after performing an infinite number of iterations, k→∞. Following Supp.Eq.4, the injected field in this k→∞ iteration would be given by the matrix $T^{\text{H}}T$ (the TRO) taken to the kth→∞ power. Thus, iTRUE is expected to converge to the eigenvector of $T^{\text{H}}T$ having the largest eigenvalue, i.e. the first singular vector of *T*.

The result of the iTRUE power-iterations, is analogous to the formation of the lowest-loss lasing mode in a laser cavity. Specifically, the result is obtained under the assumption that the input field is decomposed into all eigenvectors, V_i, of the matrix $T^{\text{H}}T$. Ordering these modes by the amplitude of their eigenvalues λ_i, such that $(T^{\text{H}}T)V_i = \lambda_i V_i$ with $\lambda_i \geq \lambda_{i-1}$, and writing the input field as $E^{\text{in}} = \sum_i a_i V_i$the injected field in the k→∞ iteration then becomes:

$$\lim_{k\to\infty}\left((T^{\text{H}}T)^{k}E^{\text{in}}\right) = \lim_{k\to\infty}\left((T^{\text{H}}T)^{k}\textstyle\sum_i a_i V_i\right) = \lim_{k\to\infty}\left(\textstyle\sum_i \lambda_i^{k} V_i\right) \propto V_1 \qquad (5)$$

Thus, for optimal optical focusing one needs to send into the medium the eigenvector V_1, of the time-reversal operator $T^{\text{H}}T$, with the largest eigenvalue, λ_1.

The process of computing the first eigenvector of $T^{\text{H}}T$ is equivalent to computing the first *singular* vector of the AOTM, *T*,. Thus, all that is required to find the optimal focusing wavefront, is to perform a singular value decomposition (SVD) of the AOTM. SVD of a matrix *T* is given by $T = USV^{*}$, where *S* is a rectangular diagonal matrix containing the real positive singular values, μ_i, in descending order, and *U* and *V* are unitary matrices whose columns corresponds to the output and input singular vectors, U_i and V_i, respectively. Each input singular vector V_i corresponds to the input field (at the SLM plane) that corresponds to the ith singular value, λ_i. The corresponding output singular vector U_i is expected resulting field at the camera plane.

Supplementary note 4: Dependence of the AOTM singular values distribution on the ultrasound focus shape

As mentioned in the manuscript, the distribution of singular values of the AOTM (Fig.1c – blue dots) is dictated by the shape of the acoustic focus and the number of optical modes contained inside the acoustic focus, and does not follow the Marčenko-Pastur distribution of the all-optical TM (Fig.1c – gray dots). The number of significant singular values is the number of optical modes (speckles) contained inside the acoustic focus, since it is the number of optical modes that decompose the virtual 'aperture' that is effectively formed by the ultrasound focus. For the Gaussian-shaped ultrasound focus considered in our simulations whose results are presented in Fig.1, the singular values gradually decrease. However, different ultrasound focus shape would result in a different distribution.
As a synthetic example and demonstration we have repeated the simulations of Fig.1 with a top-hat flat-top circular shaped tagging area, with homogenous tagging efficiency. While such a top-hat tagging with sharp edges is not achievable in practice, we consider it as a simple example for demonstrating the relationship between the ultrasound focus shape (the distribution of the pressure amplitude) and the distribution of the singular values of the AOTM. As can be seen from Supplementary Figure S3b below, the singular values in the case of the top-hat tagging abruptly decrease at the number of optical modes contained in this virtual hard aperture.
Supplementary Figure 3 presents the singular values obtained via SVD of two simulated AOTMs. The first (Supplementary Fig.3a) is an AOTM measured with a two-dimensional Gaussian shaped ultrasonic focus, close to the one expected to be achieved in practice, and the same one used to simulate the results of Figure 1 in the main text.
The second (Supplementary Fig.3a) is for a theoretical tagging ultrasound focus having a 'top-hat' circular shape, with sharp defined edges. It can be observed the singular values sharply fall off for the top-hat shaped-target, as expected[7], where the singular values for the Gaussian shaped focus fall gradually. Supplementary
Figures 4-5 display the results of focusing using the singular vectors corresponding to the first 162 singular values presented in Supplementary Figure 3, for both the Gaussian tagging beam (Supplementary Figure S4, Figure 1), and the top-hat beam (Supplementary Figure 5)

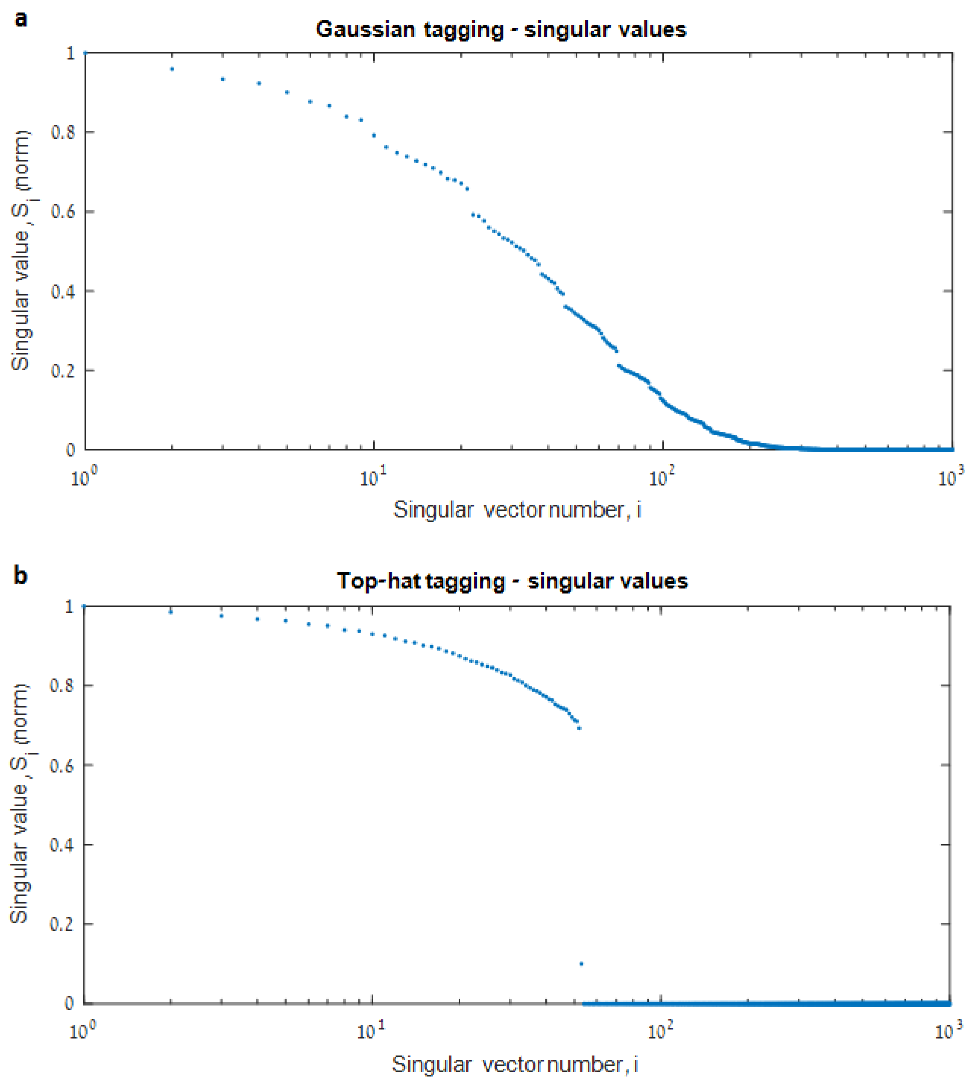

Supplementary Figure 3: Singular values of a Gaussian ultrasound focus (a) and a top-hat circular shaped tagging area (b)

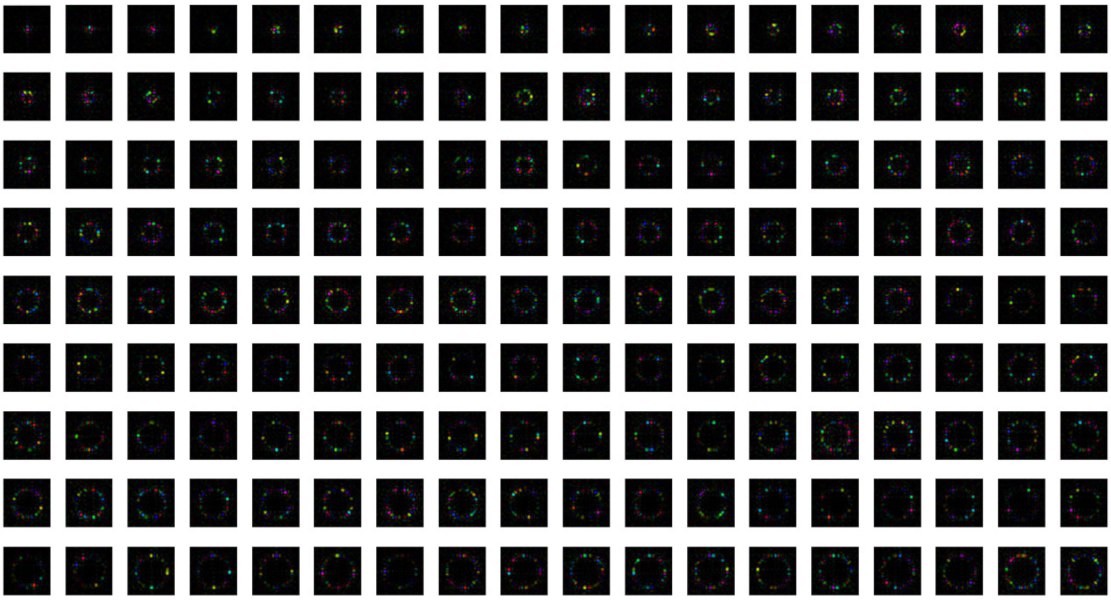

Supplementary Figure 4: Gaussian ultrasound focus singular vectors Optical fields obtained at the acoustic focal plane when focusing with first 162 singular vectors of a Gaussian tagging area

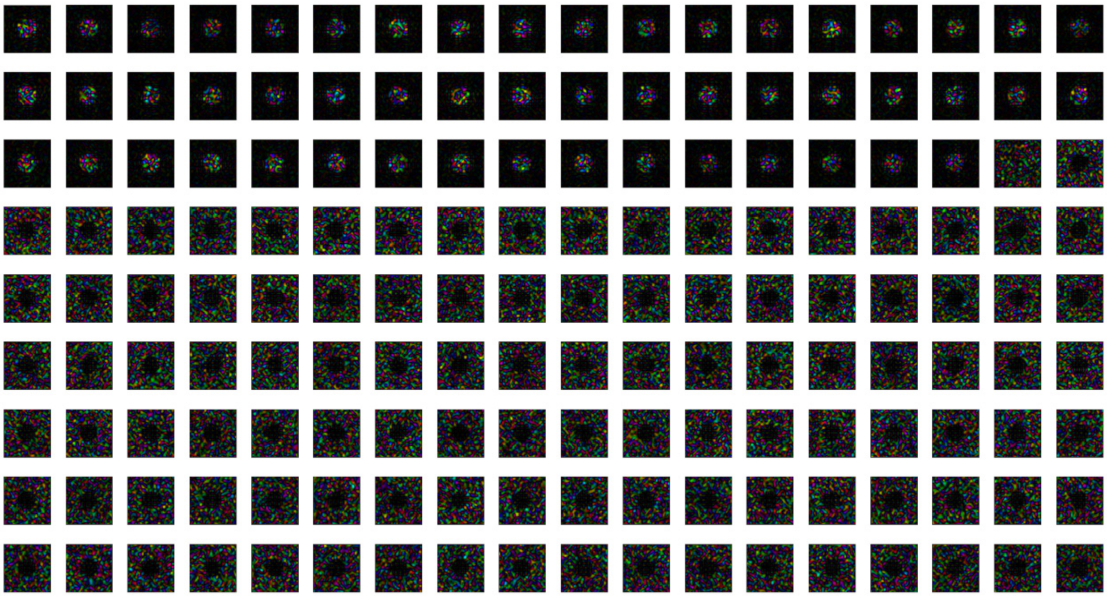

Supplementary Figure 5: Circular top-hat ultrasounf focus singular vectors Optical fields obtained at the acoustic focal plane when focusing with first 162 singular vectors of a 'top-hat' circular tagging area

Supplementary note 5: Dependence of the AOTM focus size as a function of the number of degrees of control (SLM pixels), speckle grain size, and SNR: Theoretical lower bound

As we demonstrate in our experiments, the size of the focus obtained by the injecting first singular vector of the AOTM can be considerably smaller than the ultrasound focus size, providing sub acoustic-diffraction resolution, which may reach the optical diffraction-limit. However, as we show below, the dimensions of the obtained focus depends strongly on the ratio between the ultrasound focus size and the optical speckle grain size, and on the number of controlled degrees of freedom (SLM pixels).

In this Supplementary Note we provide a basic theoretical analysis of the expected focus size as a function of the speckle grain size, number of controlled degrees of freedom (SLM pixels, N_{SLM}), and signal to noise ratio (SNR). This analysis allows to estimate the number of degrees of control (AOTM rows) that are required to reach the optical diffraction-limit, as demonstrated in our experiments, and understand the limitation of our approach (as well as other approaches for acousto-optical guided sub acoustic-diffraction focusing, such as TROVE and iTRUE, which are limited by the same factor). The basic simple derivation we provide assumes 2D Gaussian-shape tagging.

Analysis in the ideal noise-free case:

In the perfect case of noise-free measurements and infinite number of controlled and detected degrees of freedom the focus size of the first singular vector is of the optical speckle-grain dimensions. This is allowed by the fact that the small difference between the energy tagged at the center of the acoustic focus (i.e. the first singular value of the AOTM) is measurably larger than the second singular value of the AOTM. However, in practice, the smaller are the optical speckle grain dimensions (with respect to the ultrasound focus), and the larger is the measurement noise, the less discernible is the small difference between the energy tagged at the center of the ultrasound focus and the energy tagged when the optical focus is shifted $\Delta r = \sigma_{\text{speckle}}$ away from the ultrasound focus center, where σ_{speckle} is the speckle grain size. Thus, in practice, the focus size of the first singular vector is determined by the ability to distinguish small differences in ultrasonically-tagged energies between the first and second singular vectors of the AOTM.

Below we use this condition to obtain a lower bound for the focus size of the first singular vector. The condition is obtained by imposing the ultrasonically-tagged energy of light focused at a distance Δr from the center of the ultrasound focus to be distinguishably smaller than the ultrasonically-tagged energy of light focused at the center of the ultrasound focus. Specifically, by 'distinguishably smaller' we require that the energy difference between the two focal spots is larger than the natural statistical variations of the ultrasonically-tagged energy, given by the speckle statistics and the finite AOTM size, and measurement noise.

We assume an ultrasound focus spot of dimensions σ_{US}, having a Gaussian spatial pressure distribution $P_{US}(r) \propto exp\left\{-\frac{r^2}{2\sigma_{US}^2}\right\}$, which provides the ultrasonic tagging, and an optical speckle grain size of dimensions, σ_{speckle}.

We begin by estimating the optimal energy tagging of light focused at the center of the acoustic focus (the first singular vector of the AOTM) for the noise-free case: For phase-only wavefront-shaping the intensity enhancement of a single speckle grain, in the noise-less case, is given by[8]: $\eta \approx \frac{\pi}{4} N_{\text{SLM}}$. Thus, the energy tagged through the ultrasound focus when the ideal first singular vector is injected into the medium, E_1, should be the sum of the energy of an intensity enhanced speckle passing through the center of the ultrasound focus, ηE_{speckle}, and the 'background' speckles inside the ultrasound focus that are not intensity enhanced, but are ultrasonically tagged, B_1:

$$E_1 \propto \eta E_{\text{speckle}} \cdot P_{\text{US}}(r=0) + B_1 \approx \eta E_{\text{speckle}} + B_1 \qquad (6)$$

Where $E_{speckle}$ is the average energy of a single speckle grain, ηE_{speckle} is the energy of the wavefront-shaped speckle grain at the focus, and $B_1 \approx E_{\text{speckle}} N_{\text{speckles}}$, is a background term originating from the $N_{\text{speckles}} \approx \left(\frac{\sigma_{\text{US}}}{\sigma_{\text{speckle}}}\right)^2$ speckle grains that are contained in the ultrasound focus, and ultrasonically tagged.

To provide a lower bound to the dimensions of the of the first singular vector under realistic conditions, we require that the total ultrasonically-tagged energy through the ultrasound focus for the second singular vector is measurably lower than that of the first singular vector. We approximate the tagged energy of the second singular vector, E_2, by considering a wavefront-shaped intensity-enhanced speckle that is located at a transverse distance of Δr away the center of the ultrasound focus:

$$E_2 \propto \eta E_{\text{speckle}} \cdot P_{\text{US}}(r = \Delta r) + B_2 \approx \eta E_{\text{speckle}} \left(1 - \frac{\Delta r^2}{2\sigma_{\text{US}}^2}\right) + B_2 \quad (7)$$

Where in the right-hand side, the ultrasound pressure distribution was approximated by a Taylor series, assuming Δr is appreciably smaller than the ultrasound focus size.

To determine the dimensions of the focus for the first singular vector we require the ultrasonically-tagged energy by this focus, E_1, i.e. the first singular value, should be larger than the ultrasonically-tagged energy by the second singular vector, E_2, focusing at a transverse distance of Δr away the center of the ultrasound focus:

$$E_1 > E_2 \quad (8)$$

$$\eta E_{\text{speckle}} + B_1 > \eta E_{\text{speckle}} \left(1 - \frac{\Delta r^2}{2\sigma_{\text{US}}^2}\right) + B_2 \quad (9)$$

$$\eta E_{\text{speckle}} \frac{\Delta r^2}{2\sigma_{\text{US}}^2} > B_2 - B_1 \quad (10)$$

Each of the background terms B_1, B_2 in equation (10) is the sum of the N_{speckles} contained within the ultrasound focus in the given speckle realization, and can be approximated by:

$$B_i \approx E_{\text{speckle}}\left(N_{\text{speckles}} \pm \sqrt{N_{\text{speckles}}}\right) \approx E_{\text{speckle}} \left(\frac{\sigma_{\text{US}}}{\sigma_{\text{speckle}}}\right)^2 \pm E_{\text{speckle}} \left(\frac{\sigma_{\text{US}}}{\sigma_{\text{speckle}}}\right) \quad (11)$$

Where the $\pm\sqrt{N_{\text{speckles}}} = \pm\sqrt{\left(\frac{\sigma_{\text{US}}}{\sigma_{\text{speckle}}}\right)^2}$ is the statistical fluctuations in the total energy of the N_{speckles} background speckles, arising from the statistics of fully developed speckles, i.e. exponential intensity statistics with a variance equal to the mean intensity.

Plugging (S11) into (S10) yields the condition:

$$\eta E_{\text{speckle}} \frac{\Delta r^2}{2\sigma_{\text{US}}^2} >\sim \sqrt{2} E_{\text{speckle}} \left(\frac{\sigma_{\text{us}}}{\sigma_{\text{speckle}}}\right) \quad (12)$$

Isolating the Δr term, i.e. the radii of the formed focus, and plugging $\eta = \frac{\pi}{4} N_{\text{SLM}}$. leads to:

$$\left(\frac{\Delta r}{\sigma_{\text{us}}}\right)^2 >\sim \frac{8\sqrt{2}}{\pi N_{\text{SLM}}} \left(\frac{\sigma_{\text{US}}}{\sigma_{\text{speckle}}}\right) \quad (13)$$

Providing the final result of the focus radius in the noise-free case:

$$\Delta r >\sim \sqrt{\frac{8\sqrt{2}}{\pi N_{\text{SLM}}} \left(\frac{\sigma_{\text{US}}}{\sigma_{\text{speckle}}}\right)}\, \sigma_{\text{US}} \approx \sqrt{\frac{3.6}{N_{\text{SLM}}} \frac{\sigma_{US}}{\sigma_{\text{speckle}}}}\, \sigma_{\text{US}} \quad (14)$$

For example: for our experimental parameters, with large speckle grains: $\frac{\sigma_{\text{US}}}{\sigma_{\text{speckle}}} \approx 6$, and $N_{\text{SLM}} = 3{,}500$, the lower bound for the focus diameter , $2\Delta r$, is:

$$2\Delta r >\sim \frac{1}{6.11} \sigma_{\text{US}} = 1.06 \cdot \sigma_{\text{speckle}}$$

Which is in accordance with the obtained single speckle grain scale focus

For the more realistic case of diffraction-limited speckles and a high frequency (>50MHz) ultrasound transducer, one can plug $\frac{\sigma_{\mathrm{US}}}{\sigma_{\mathrm{speckle}}} \approx 50$. In this case, for a realistically large number of degrees of control of $N_{SLM} = 10{,}000$, the expected lower bound on the obtained focus diameter for the first singular vector of the AOTM is:

$$2\Delta r >\sim 0.27\sigma_{\mathrm{US}} \approx 13.4 \cdot \sigma_{\mathrm{speckle}}$$

Which while is about 3.7 times below the ultrasound focus size, is considerably larger than the speckle-grain diffraction limited dimensions.

In Supplementary Figure 6 we plot the bound on the obtained focus diameter as a function of N_{SLM} for several ratios of ultrasound focus size to speckle grain size. We confirm the validity this basic theoretical estimation for various speckle grain sizes, N_{SLM}, and SNR in Supplementary Note 6 below, by performing a large set of numerical simulations.

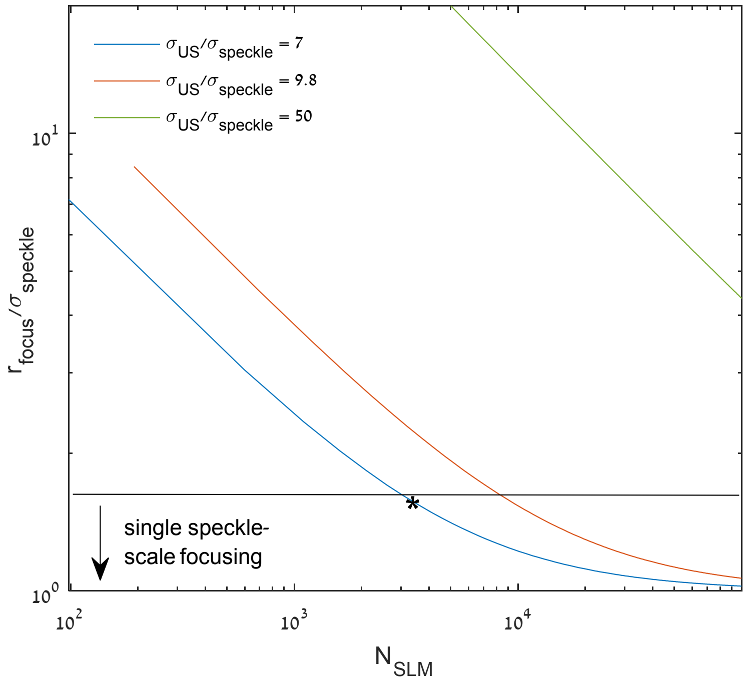

Supplementary Figure 6: Theoretical lower bound for the focal spot size obtained using the first singular vector of the AOTM in the noise-less case, using 10,000 output modes (camera pixels). The plotted focal spot size is estimated by $r_{\mathrm{focus}} = \sqrt{\sigma_{\mathrm{speckle}}^2 + (2\Delta r)^2}$, where Δr is taken from Supplementary Equation 14. The asterisk (*) marks the parameters of our experiments with single AOTM focusing.

Condition for single-speckle grain focusing:

The result of Supplementary Equation (14) for the noise-less case can be used to estimate the number of degrees of control that is required to get a focus size of the optical diffraction limit dimensions (a single speckle grain). For estimating this value we plug $\Delta r < \sigma_{\mathrm{speckle}}/2$ in (14) to get:

$$\frac{\sigma_{\mathrm{speckle}}}{2} >\sim \sqrt{\left(\frac{3.6}{N_{\mathrm{SLM}}}\frac{\sigma_{\mathrm{us}}}{\sigma_{\mathrm{speckle}}}\right)}\sigma_{\mathrm{us}} \qquad (15)$$

$$N_{\mathrm{SLM}} >\sim 14.4\left(\frac{\sigma_{\mathrm{us}}}{\sigma_{\mathrm{speckle}}}\right)^3 \qquad (16)$$

This result suggests that the number of required controlled input modes (number of columns in the AO-TM) is expected to scale cubically with the ratio between the ultrasound focus diameter and speckle

diameter. For example, for the situation considered in our experiments: $\frac{\sigma_{\mathrm{US}}}{\sigma_{\mathrm{speckle}}} \approx 6$, $N_{\mathrm{SLM}} > 3100$ is required, in agreement with the value required in our experiments.

Obtained focus size in the finite SNR case:

The above derivation assumed noise-free measurements. Measurement noise may be considered by changing the intensity enhancement to $\eta = \frac{\pi}{4} N_{SLM}(1 - NSR^2)$, where $NSR = (SNR)^{-1}$ is the noise-to-signal ratio[9], and adding an additional noise term $n \approx \frac{NSR}{\sqrt{M}} \cdot E_{\mathrm{speckle}}(\sigma_{\mathrm{US}}/\sigma_{\mathrm{speckle}})^2$ to the right-hand side of equation (12), where M is the number of measurements used to determine the ultrasonically-tagged energy:

$$\eta E_{\mathrm{speckle}} \frac{\Delta r^2}{2\sigma_{\mathrm{US}}^2} > (B_2 - B_1) + n \qquad (16)$$

$$\eta E_{\mathrm{speckle}} \frac{\Delta r^2}{2\sigma_{\mathrm{US}}^2} > \sqrt{2} E_{\mathrm{speckle}} \left(\frac{\sigma_{\mathrm{US}}}{\sigma_{\mathrm{speckle}}}\right) + \frac{NSR}{\sqrt{M}} \cdot E_{\mathrm{speckle}} \left(\frac{\sigma_{\mathrm{US}}}{\sigma_{\mathrm{speckle}}}\right)^2 \qquad (17)$$

$$\Delta r >\sim \sqrt{\frac{3.6}{N_{\mathrm{SLM}}(1-NSR^2)} \left(\frac{\sigma_{\mathrm{US}}}{\sigma_{\mathrm{speckle}}} + \frac{NSR}{\sqrt{2M}} \left(\frac{\sigma_{\mathrm{US}}}{\sigma_{\mathrm{speckle}}}\right)^2\right)}\, \sigma_{\mathrm{US}} \qquad (18)$$

Measurement noise thus affects the focus size by two terms: the first is the lower enhancement, which interestingly, even for SNR as low as SNR=3, is reduced only by a factor of $(1 - NSR^2) \approx 0.9$. The second is the second term in (S18), where the finite SNR becomes non-negligible when: $\frac{NSR}{\sqrt{2M}} \left(\frac{\sigma_{\mathrm{US}}}{\sigma_{\mathrm{speckle}}}\right)^2 > \frac{\sigma_{\mathrm{US}}}{\sigma_{\mathrm{speckle}}}$, i.e. when: $SNR < \frac{1}{\sqrt{2M}} \frac{\sigma_{\mathrm{US}}}{\sigma_{\mathrm{speckle}}} = \sqrt{\frac{N_{\mathrm{speckles}}}{2M}}$. This suggests a higher SNR is required in the case of smaller optical speckle grain size (compared to the ultrasound focus dimension).

It is important to note that the above analysis for the obtained focus dimensions with and without noise considers the case of focusing using a single AOTM. Using two or more AOTMs for focusing is expected to provide smaller focus size, and is advantageous over focusing using a single AOTM, since the decomposition of several AOTMs provides an effective virtual acoustic focus that is sharper than each of the individual, acoustic-diffraction limited foci, as is visible in the experimental results of Figure 4(c-h).

Supplementary note 6: Dependence of the AOTM focus size as a function of the number of degrees of control (SLM pixels), and speckle grain size, and SNR: Numerical investigation

The theoretical analysis in Supplementary Note 5 for the obtained focus size and the number of measurements (input modes) required for single speckle scale focusing, provide theoretical upper and lower bounds, for these, respectively. To validate and to better investigate the actual values expected in experiments under different conditions, we have performed a large set of numerical simulations with different ultrasound focus to speckle grain size dimensions, and various SNRs. The results of these investigations are presented in Supplementary Figures 7-8. The two most important goals of these studies was to compare to the theoretical and numerical values to the ones observed in our proof-of-principle experiments, and to project the values expected in experiments with diffraction-limited speckles. The latter is important for the use of the AOTM (and similarly TROVE) in practical imaging and focusing applications deep inside scattering samples, where the speckle grain size is expected to be of diffraction-limited dimensions, i.e. an order of magnitude smaller, compared to the ultrasound focus size, than in our proof-of-principle experiments or similar experiments in recent works.

Supplementary Figure 7 provides the results for focusing in the absence of noise, for small and intermediate ratios between the ultrasound focus size and the speckle grain size (i.e. for relatively large speckle grains), as is used in our experiments and in the demonstration of TROVE. As can be observed from the simulations results of Supplementary Figure 7.c, taking into account that the experiments of Figure 2 were obtained with an ultrasound focus size of $\sigma_{US}\approx 6.5\sigma_{speckle}$, the simulation results for $\sigma_{US}\approx 7\sigma_{speckle}$ (bold black line) indicate that N_{SLM}>~3,000 is required to achieve near single speckle grain focusing, closely fitting the number of input modes that was required for single speckle grain focusing in our experiments. Importantly, since the AOTM SVD focusing approach is mathematically equivalent to maximizing the speckle variations in the TROVE approach (though they originate from different fundamental considerations) the number of sequential measurements required in the AOTM approach is the same as the number of measurements required in TROVE. In our experiments, taking into account measurement noise and instabilities, we chose to experimentally measure the maximum number of modes possible in the minutes-scale decorrelation times of our samples and setup. We note that a similar limitation exists on the minimum number of output modes recorded, as an $N\times M$ matrix has a rank that is limited by the minimum of N or M. In practice, the acquisition time posed a limit on the number of measured input modes, since, unlike the output modes, they are sequentially measured.

The diameters of the obtained foci in Supplementary Figure 7 were calculated by standard deviation of the radius of the obtained intensity distribution thresholded at $1/8^{th}$ its peak intensity, to reduce the background effects on the calculated diameters.

Supplementary Figure 8 presents the results of the study of obtained focus size for smaller speckle grains, compared to the speckle-grain to ultrasound diameter used in our experiments, under the presence of various levels of measurement noise. The numerical results are in accordance with the theoretical estimates of Supplementary Figure 6, as they show that for a reduced speckle grain size, the focusing diameter becomes larger, unless a larger number of input modes is measured. The limitation on the experimental measurement time posed by sample decorrelation in most practical applications, and the requirement for sequential measurements in the presented approach, thus limit the resolution increase possible by the AOTM (and TROVE), in practical scenarios with diffraction-limited speckles.

The chosen ratio of ultrasound focus to speckle grain size of $\frac{\sigma_{US}}{\sigma_{speckle}} = 50$, reflects an optimistic and desired case of high-frequency (e.g. 90MHz) ultrasound focus, and long-wavelength (e.g. 1064nm) speckles. In case of lower ultrasound modulation frequencies, the focusing performance are expected to degrade accordingly.

The diameters of the obtained foci in Supplementary Figure 8 were calculated by a Gaussian fit to the obtained foci, ultrasound focus, and a single speckle grain.

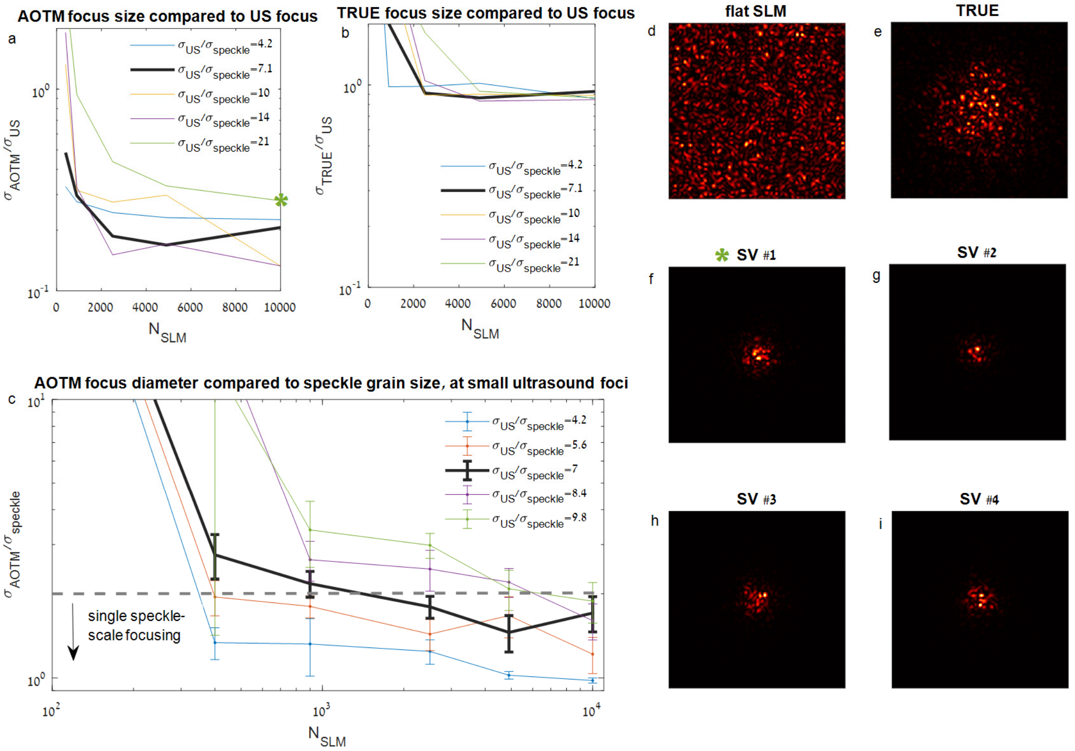

Supplementary Figure 7: Simulation results for focal spot size obtained with the AOTM in the noise-less case using 10,000 output modes (camera pixels), compared to TRUE focusing: a. AOTM focus spot diameter (σ_{AOTM}) as a function of N_{SLM}, for different ultrasound focus diameter (σ_{US}=*4.2* to *21* speckle grains diameter, $\sigma_{speckle}$); Vertical scale is normalized to the ultrasound focus diameter size, σ_{US}; b. Same as (a) for TRUE focusing, showing the acoustic diffraction limitation; c. Same as (a) for smaller ultrasound foci, average over 3 different realizations of the scattering medium. Vertical scale is in speckle grain size units (σspeckle), single speckle focusing is obtained for $\sigma_{AOTM}/\sigma_{speckle}<\sim 2$. (d-i) Example of the obtained intensity distribution at the ultrasound focal plane for a single realization of the largest simulated ultrasound focus, having a diameter of *~21.3* speckle grains, i.e. containing *~450* speckle grains (marked by * in (a)), for: d. flat SLM; e. TRUE focusing; f-i, injecting the first four singular vectors of the AOTM measured with N_{SLM}=*10,000*. The experiments of Figure 2 were obtained with an ultrasound focus size of $\sigma_{US}\approx 6.5\sigma_{speckle}$. The simulation results for $\sigma_{US}\approx 7\sigma_{speckle}$ are given by the bold black line in (a-c). To achieve single speckle grain focusing in these conditions $N_{SLM}>\sim 2,500$ is required. Error bars, standard deviations of three different realizations.

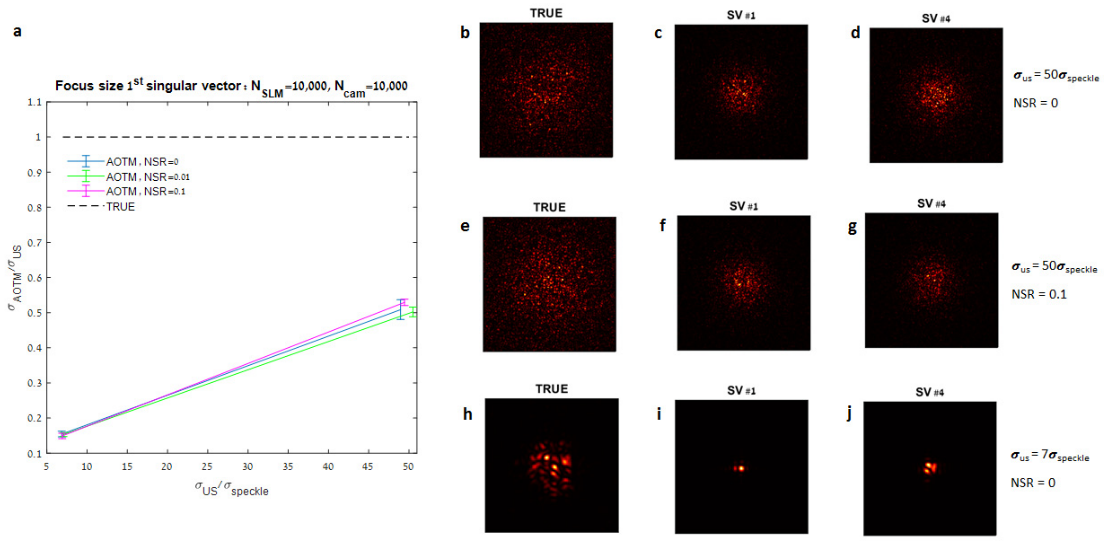

Supplementary Figure 8: Numerically obtained focus size in the case of finite SNR and small speckle grain: **a.** AOTM focus spot diameter (σ_{AOTM}) as a function of the ratio between the ultrasound focus and the speckle grain size ($\sigma_{US}/\sigma_{speckle}$), for different noise-to-signal (NSR) ratios. **(b-d)** Comparison of the intensity distribution at the focus for the case of $\sigma_{US}/\sigma_{speckle}=50$ between TRUE focusing (b), and the first (c) and fourth (d) singular vectors of the AOTM, for *NSR=0;* **(e-g)** same as (b-d) for *NSR=0.1*; **(h-j)** same as (e-g) for $\sigma_{US}/\ \sigma_{speckle}=7$. Error-bars, standard deviations of three different realizations.

Supplementary note 7: Numerical investigation of the expected peak-to-background ratio (PBR)

In order to study the expected intensity enhancements, i.e. the peak to background ratio (PBR), of the obtained foci with the AOTM technique vs. those expected with TRUE focusing, we have performed additional simulations similar to those performed to produce Supplementary Figure 7. The results of these investigations are displayed in Supplementary Figure 9. The results present the PBR of AOTM focusing vs. the PBR of TRUE focusing as a function of the number of SLM pixels, camera pixels, and ultrasound focus to speckle grain size dimensions. The results are in line with the theoretical predictions for PBR≈N_{SLM}/N_s, where N_s is the number of speckle grains contained in the formed focus (N_s is approximately $N_s \approx (\sigma_{US}/\sigma_{speckle})^2$ for TRUE focusing, and lower for AOTM focusing).

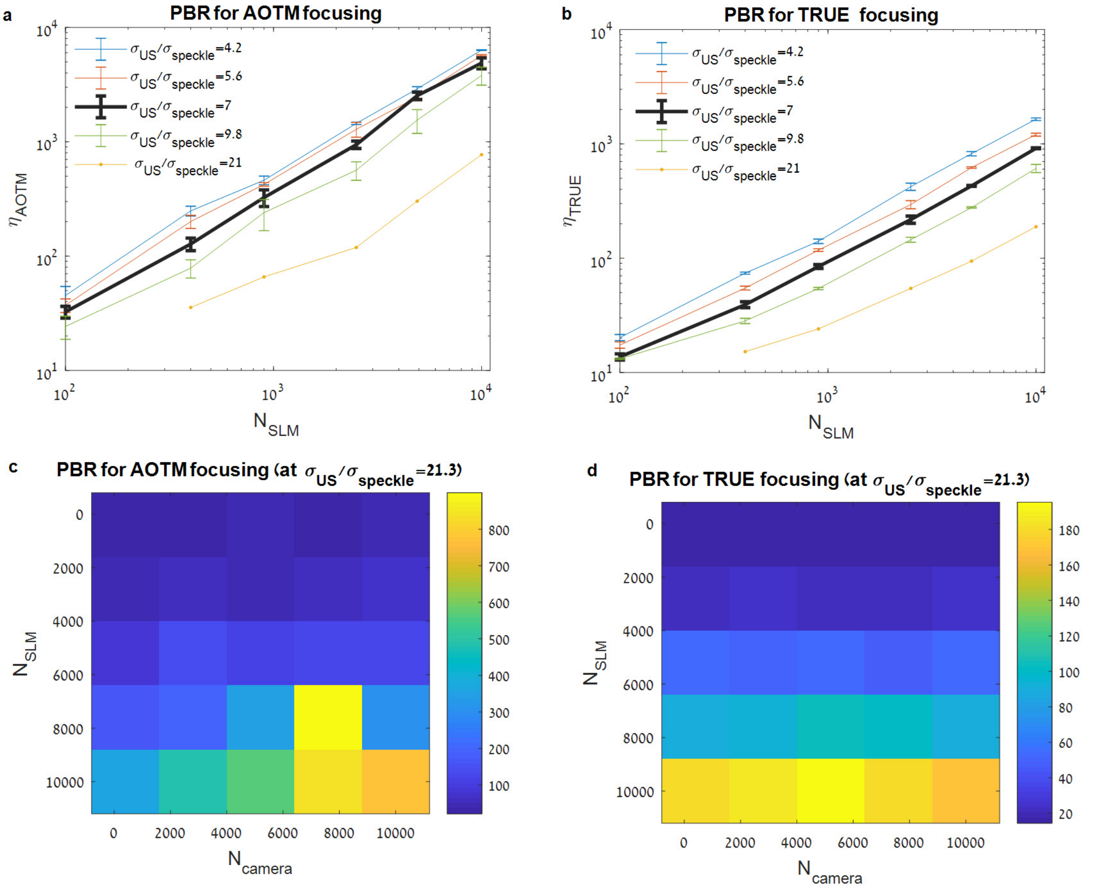

Supplementary Figure 9: Numerical investigation of the peak-to-background ratio (PBR) of AOTM and TRUE focusing as a function of the number of AOTM input modes (number of SLM pixels, N_{SLM}), and output modes (camera pixels, N_{camera}), for different ultrasound focus size**. a.** PBR for AOTM focusing using N_{camera}=*10,000*; **b,** same as (a) for TRUE focusing; **c,** PBR dependence of AOTM focusing on N_{SLM} and N_{camera} for an ultrasound focus spot diameter of *21.3* speckle grains; **d**, same as (c) for TRUE focusing. Error bars, standard deviation over three different realizations.

Supplementary note 8: Example of an experimentally measured AOTM

In Supplementary Figure 10 we display the measured AOTM in the experiment described in Fig.2, alongside with the normalized values of its first 1,000 singular vectors, and the measured ultrasound modulated field for the first input Hadamard vector.

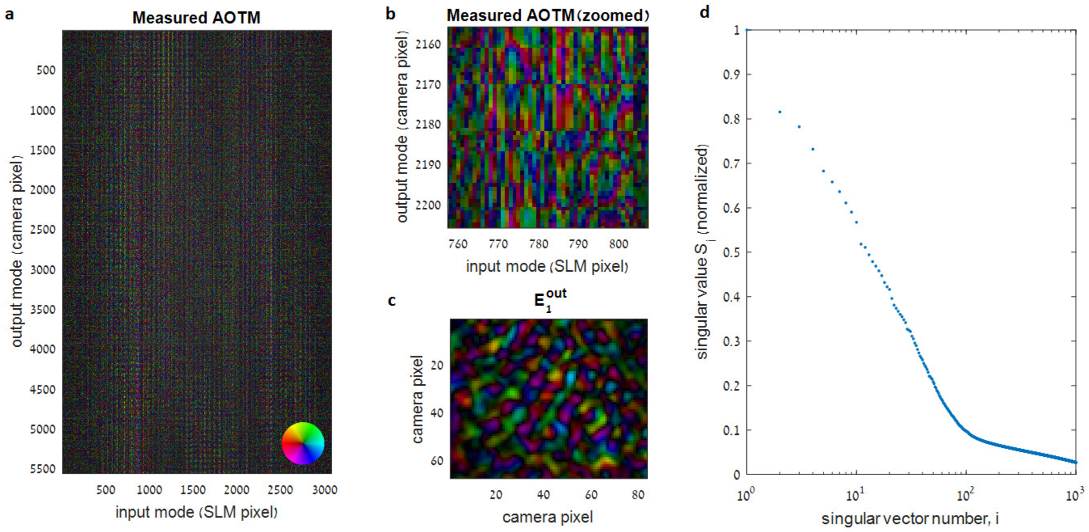

Supplementary Figure 10: Experimentally measured AOTM in the experiment of Fig.2: a, Full measured AOTM with having *3072* input modes and *5560* output modes (bottom-right inset: color-map used for displaying the complex field amplitude and phase). **b,** zoom-in on a small fraction of the AOTM displayed in (a), spatial correlations are observed due to intentional oversampling of the output speckle grain dimensions. **c,** an example for the output ultrasound modulated field measured for the first input Hadamard vector, **d,** normalized values of the first *1,000* singular vectors of the measured AOTM.

While the scattering matrix of a single thin scattering layer should have sparsity in the appropriate transform basis[10], as result of memory-effect correlations, in the experimental configuration where we have two successive layers at a large distance from each other, and a small region is ultrasonically tagged in between the scattering layers, The resulting AOTM has very low sparsity - as seen in the revised Figure 2, above. The phase and amplitude correlations that can be observed in the AOTM columns (output modes) are expected due to the oversampling of the speckle output modes by the camera pixels, as can be seen in the example for a single measured output field at the camera plane in Supplementary Figure 8(c).

The singular values distribution of the experimentally measured AOTM (Supp. Fig.10(d)) follows the distribution obtained from the numerical simulated AOTM of Fig.1c, which models the case of diffusive multiple-scattering light propagation in a thick sample.

Supplementary Note 9: Number of measurements required in TRUE, iTRUE, TROVE and the AOTM

In order to provide a comparison between the different state of the art acousto-optic focusing techniques, namely TRUE, iTRUE, TROVE and the AOTM, we have used the numerical results of Supplementary Figure 7, for the obtained focus size, which were performed for a single ultrasound focus AOTM, to form the comparison table presented in Supplementary Table 1. This table summarizes the estimated number of measurements required to achieve optically diffraction-limited focus and scan it inside the ultrasound focus area, using the different approaches. In general, while it is not possible to achieve optical diffraction-limited focusing in TRUE, this is in principle possible in all of the other approaches. Considering that the singular value decomposition of the AOTM, which is the analogue of the all-acoustic DORT technique[11], is in fact the same approach as TROVE, both the AOTM and TROVE technique require the same number of measurements. iTRUE requires a number of iterations (=measurements) that is equal to the number of speckles contained in the ultrasound focus, for single speckle grain focusing. This number is the square of the ratio between the ultrasound focus diameter and the optical speckle grain diameter: $N_{\text{iterations}}=(\sigma_{\text{US}}/\sigma_{\text{speckle}})^2$. However in iTRUE, only a single focus is formed, and in order to perform scanning, the same number of iterations has to be repeated for every point within the ultrasound focus, thus the number of measurements will be $N_{iterations}(\sigma_{\text{US}}/\sigma_{\text{speckle}})^2=(\sigma_{\text{US}}/\sigma_{\text{speckle}})^4$. In TROVE/AOTM when multiple ultrasound foci (e.g. four foci) are used, the number of measurements required for focus scanning is simply the number of measurement required for single focus times the number of ultrasound foci. Taking into account that using four ultrasound foci allows, in principle, scanning over a field of view that is may be four times larger than the single ultrasound focus, the approximate number of measurement required for focus scanning in TROVE/AOTM with multiple ultrasound foci is approximately the same as the number of measurements required to achieve a single focus.

Interestingly, the results we have obtained from our simulations for the number of measurements required using a single ultrasound focus show that the total number of measurements required in TROVE/AOTM in the specific parameters considered is comparable to the number of measurements required in iTRUE. It is important to note that this is by no means a general conclusion, and is only a numerically obtained estimate with a single ultrasound focus. As is shown in Figure 4, the focus formed by joint decomposition using multiple ultrasound foci is sharper than the one obtained with a single focus, and we thus expect that TROVE/AOTM will outperform iTRUE in the specific parameters range that was numerically investigated. Additionally, AOTM and TROVE may gain from possible parallelization of multiple foci measurements, e.g. by different ultrasound carrier frequencies or other coding mechanism, such as chirped pulses[12], or plane waves[13].

**Number of measurements required for single diffraction-limited focus:**				
	TRUE	iTRUE	TROVE/AOTM with a single ultrasound focus	TROVE/AOTM with four ultrasound foci
$\sigma_{\text{US}}\approx 4.2\sigma_{\text{speckle}}$	N.A.	18	~300	~300
$\sigma_{\text{US}}\approx 7\sigma_{\text{speckle}}$	N.A.	49	~2,000	~2,000
$\sigma_{\text{US}}\approx 10\sigma_{\text{speckle}}$	N.A.	100	~10,000	~10,000
**Number of measurements required for scanning all points inside an acoustic focus**				
$\sigma_{\text{US}}\approx 4.2\sigma_{\text{speckle}}$	N.A.	$\sim 4.2^2 \text{x} 4.2^2 = \sim 300$	$\sim 300 \text{x} 4.2^2$	<~300
$\sigma_{\text{US}}\approx 7\sigma_{\text{speckle}}$	N.A.	~49x49 = ~2400	$\sim 2{,}000 \text{x} 7^2$	<~2,000
$\sigma_{\text{US}}\approx 10\sigma_{\text{speckle}}$	N.A.	~100x100 = ~10,000	$\sim 10{,}000 \text{x} 10^2$	<~10,000

Supplementary Table 1: Comparison of the number of measurements required in the different acousto-optic guided focusing approaches

Supplementary references

1. Atlan, M., Forget, B.C., Ramaz, F., Boccara, A.C. & Gross, M. Pulsed acousto-optic imaging in dynamic scattering media with heterodyne parallel speckle detection. *Optics Letters* **30**, 1360-1362 (2005).
2. Gross, M. & Atlan, M. Digital holography with ultimate sensitivity. *Optics letters* **32**, 909-911 (2007).
3. Si, K., Fiolka, R. & Cui, M. Breaking the spatial resolution barrier via iterative sound-light interaction in deep tissue microscopy. *Sci. Rep.* **2** (2012).
4. Ruan, H., Jang, M., Judkewitz, B. & Yang, C. Iterative Time-Reversed Ultrasonically Encoded Light Focusing in Backscattering Mode. *Sci. Rep.* **4** (2014).
5. Prada, C. & Fink, M. Eigenmodes of the time reversal operator: A solution to selective focusing in multiple-target media. *Wave Motion* **20**, 151-163 (1994).
6. Tanter, M., Thomas, J.L. & Fink, M. Time reversal and the inverse filter. *J Acoust Soc Am* **108**, 223-234 (2000).
7. Komilikis, S., Prada, C. & Fink, M. in Ultrasonics Symposium, 1996. Proceedings., 1996 IEEE, Vol. 2 1401-1404 (IEEE, 1996).
8. Vellekoop, I.M. & Mosk, A.P. Focusing coherent light through opaque strongly scattering media. *Opt Lett* **32**, 2309-2311 (2007).
9. Yılmaz, H., Vos, W.L. & Mosk, A.P. Optimal control of light propagation through multiple-scattering media in the presence of noise. *Biomedical optics express* **4**, 1759-1768 (2013).
10. Judkewitz, B., Horstmeyer, R., Vellekoop, I.M., Papadopoulos, I.N. & Yang, C. Translation correlations in anisotropically scattering media. *Nat Phys* **11**, 684-689 (2015).
11. Prada, C., Manneville, S., Spoliansky, D. & Fink, M. Decomposition of the time reversal operator: Detection and selective focusing on two scatterers. *The Journal of the Acoustical Society of America* **99**, 2067-2076 (1996).
12. Forget, B.-C., Ramaz, F., Atlan, M., Selb, J. & Boccara, A.-C. High-contrast fast Fourier transform acousto-optical tomography of phantom tissues with a frequency-chirp modulation of the ultrasound. *Applied Optics* **42**, 1379-1383 (2003).
13. Laudereau, J.-B., Grabar, A.A., Tanter, M., Gennisson, J.-L. & Ramaz, F. Ultrafast acousto-optic imaging with ultrasonic plane waves. *Optics Express* **24**, 3774-3789 (2016).
